# Supplementary material for: FAIRification of computational models in biology
Source: bioRxiv. 2025 Mar 24:2025.03.21.644517. Preprint. [Version 1] doi: 10.1101/2025.03.21.644517 (PMC11974689; doi:10.1101/2025.03.21.644517)

# **SupplementaryFile_2: Visualization of FAIR assessment results for all models included in Table 1**

The result illustration is made using the FAIR-Viz tool (<https://faircombine.streamlit.app/>, github: <https://github.com/matthiaskoenig/fair-ca-visualization>). Legend: Findability (blue), Accessibility (orange), Interoperability (green), Reusability (red).


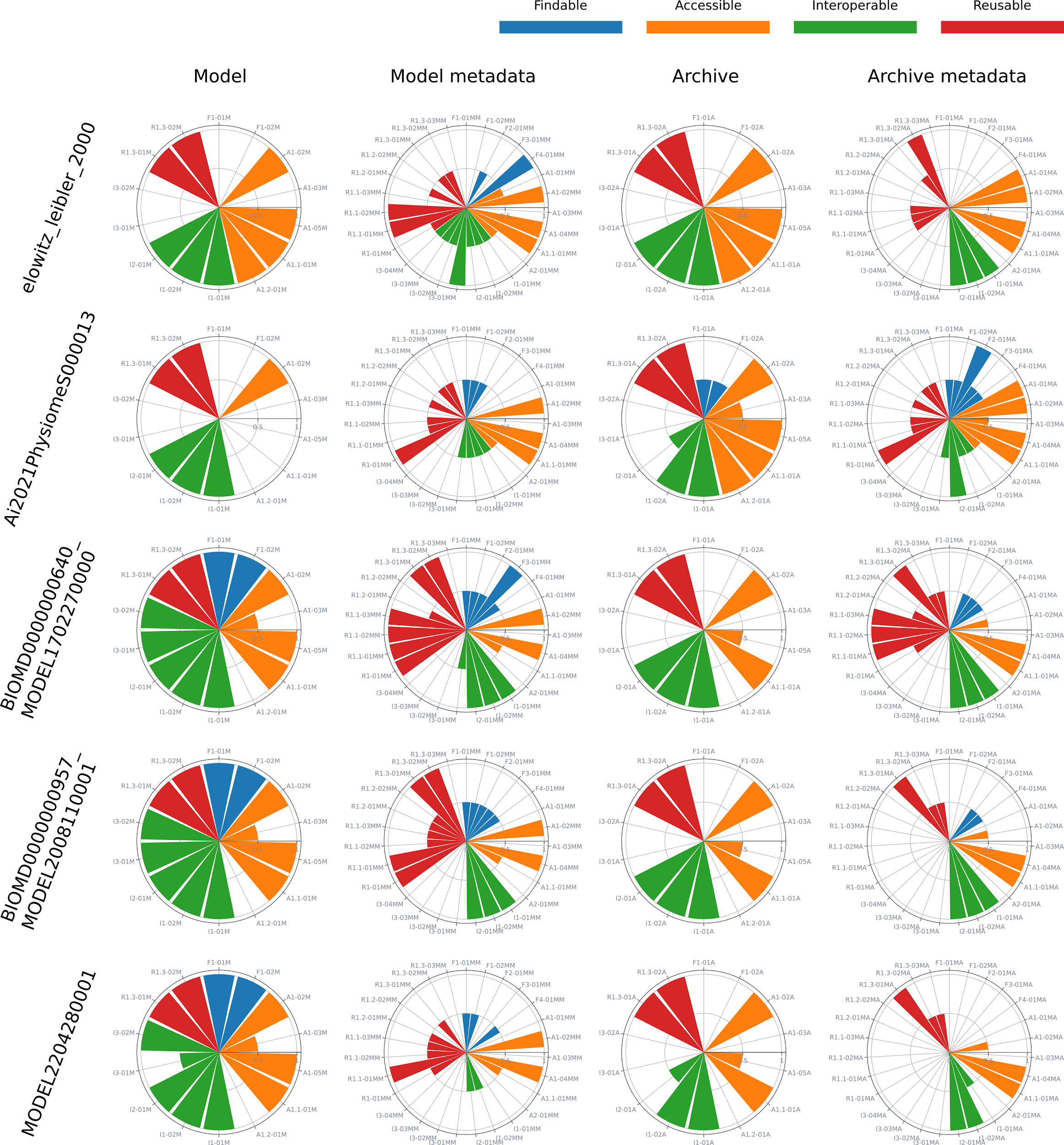

Supplement: Supplement 2 [file media-2.docx]
